# A honeycomb glomerular mesangial appearance on ultrastructural examination with minimal IgA deposition in a case of hepatic glomerulosclerosis: a case report

**DOI:** 10.12860/jnp.2014.24

**Published:** 2014-10-01

**Authors:** Ana Paula Giotto, Belén Barrón, Ana B de Diller, Alicia Torres, Marcelo Orías, Jorge Mukdsi

**Affiliations:** ^1^Servicio de Nefrología. Sanatorio Allende, Córdoba, Argentina; ^2^Servicio de Anatomía Patológica, Hospital Privado, Córdoba, Argentina; ^3^Centro de Microscopía Electrónica, Facultad de Ciencias Médicas, Universidad Nacional de Córdoba, Argentina

**Keywords:** Hepatic glomerulosclerosis, Ultrastructure, Kidney

**Implication for health policy/practice/research/medical education:**

Hepatic glomerulosclerosis is a rare condition which should be considered in patients with cirrhosis, typically alcoholic and renal manifestations.

## 1. Case

**Figure 1:**
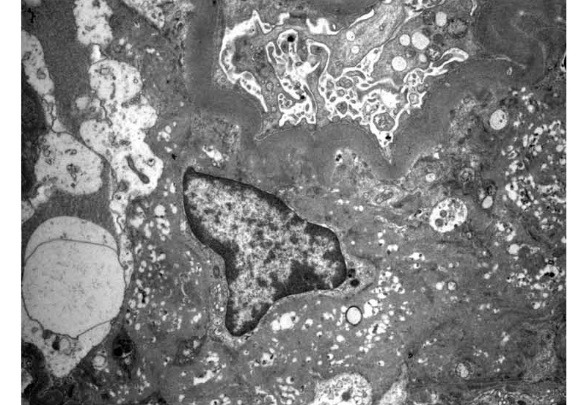
Ultrastructure of mesangial region. Original magnification 7000X.

A 63-years-old male patient with medical history of alcoholic cirrhosis after renal transplant plan was referred to our institution. Laboratory investigations demonstrated serum creatinine of 2 mg/dL, urea 89 mg/dL, potassium 4.9 mEq/L, and hemoglobin 9.7 g/dL. The complete blood count (CBC) was within the reference range and the urinalysis showed; hematuria, 5 RBCs/high power field (HPF), pyuria,15 leukocytes/ HPF and negative hemoglobin. Physical examination revealed unremarkable results: the patient’s blood pressure was 100/60 mm Hg, heart rate 65 bpm and he had ascites without edema. Renal ultrasound demonstrated normal echogenicity. A percutaneous kidney biopsy was performed. The ultrastructural examination revealed marked mesangial matrix expansion, with numerous lipid vacuoles in rounded lucencies of 150 nm, giving a honeycomb mesangial appearance (Figure 1). Few structures of a similar nature were observed in the glomerular basement membranes which appeared thickened and wrinkled, as well. The endothelium appeared swollen, occluding some capillary lumens and the podocyte foot process effacement was prominent.

On the other hand, light microscopy of the paraffin sections showed 20% of global glomerulosclerosis and immunofluorescence studies for IgG, IgA, IgM, C3, C1q and κ and λ light chains, only exhibited minimal deposition of IgA in the glomeruli. Tubular basement membranes and interstitium did not exhibit any immunoreactivity.

## 2. Discussion

Bloodwort and Sommers suggested, in 1959, that this change was not a type of usual nephritis. However, there was a specific glomerular alteration in liver cirrhosis which they named as cirrhotic glomerulosclerosis (1).

Our patient had an interesting presentation at ultrastructural level because most publications have reported this morphological pattern associated with prominent IgA deposition and only one out of 65 cirrhotic patients who underwent transvenous renal biopsy presented hepatic glomerulosclerosis without diabetes or IgA deposits (2,3). Hepatic glomerulosclerosis (HGS) has been observed in adult and children patients, as well (4). In spite of well-preserved renal function, Crawford et al. have described patterns of glomerular involvement such as minor glomerular abnormalities, HGS, membranoproliferative glomerulonephritis, and IgA nephropathy in non-alcoholic patients who underwent orthotopic liver transplantation (5). Classically, immunofluorescence in HGS reveals granular deposits of immunoglobulins and complement in glomerular capillary walls and/or the mesangium and IgA (IgA1) staining is no more intense than for the other immunoglobulins (6). An important ultrastructural differential diagnosis is lecithin cholesterol acyltransferase deficiency (LCAT) (7). In our case, a history of cirrhosis, typically alcoholic, is found in HGS and the patient lacked the systemic findings and family history characteristic of LCAT deficiency. An increase in type IV collagen, laminin and fibronectin was observed in expanded glomerular mesangial areas and along glomerular capillary walls in HGS and authors concluded that hyperproduction and/or infiltration of glomerular extracellular matrix components and interstitial collagen is closely linked to the progression of glomerular sclerosis in patients with liver diseases (8).

## 3. Conclusions

Hepatic glomerulosclerosis is a rare condition which should be considered in patients with cirrhosis, typically alcoholic and renal manifestations.

## Authors’ contributions

All authors wrote the paper equally.

## Conflict of interests

The authors declared no competing interests.

## Funding/Support

None.
